# Adolescents with worse levels of oral health literacy have more cavitated carious lesions

**DOI:** 10.1371/journal.pone.0225176

**Published:** 2019-11-27

**Authors:** Laio da Costa Dutra, Larissa Chaves Morais de Lima, Érick Tássio Barbosa Neves, Monalisa Cesarino Gomes, Luíza Jordânia Serafim de Araújo, Franklin Delano Soares Forte, Saul Martins Paiva, Fernanda Morais Ferreira, Ana Flávia Granville-Garcia

**Affiliations:** 1 Graduate Program in Dentistry, State University of Paraiba (UEPB), Campina Grande, Paraiba, Brazil; 2 Graduate Program in Dentistry, Federal University of Paraiba (UFPB), Joao Pessoa, Paraiba, Brazil; 3 Graduate Program in Dentistry, Federal University of Minas Gerais (UFMG), Belo Horizonte, Minas Gerais, Brazil; School of Dentistry, University of Sao Paulo, BRAZIL

## Abstract

The aim of the present study was to investigate whether the ability to recognize and read oral health terms is associated with the number of teeth with cavitated carious lesions in adolescents. A population-based cross-sectional study was conducted involving a sample of 746 adolescents representative of students aged 15 to 19 years at the public and private school systems in a city in northeast Brazil. Two examiners who had undergone a training and calibration exercise (inter-examiner and intra-examiner Kappa coefficient: 0.87 to 0.93) performed the diagnosis of caries using the Nyvad Index and evaluated the level of OHL (BREALD-30) of the adolescents. The participants answered questions regarding their history of visits to the dentist and the parents/caregivers answered a questionnaire addressing socioeconomic characteristics. A directed acyclic graph was created to direct the selection of covariables for adjustments in the Poisson multiple regression analysis to test the association between dental caries and OHL (α = 5%). Cavitated carious lesions (codes 3 to 6 on the Nyvad index) were found in 41.6% of the adolescents. Only 29.4% had a high level of OHL (BREALD-30 scores between 23 and 30); 42.3% of the families belonged to the A-B social class and 93% of the adolescents had been to the dentist at least once in their lifetimes. In the multivariate analysis, adolescents with inadequate (PR: 1.69; 95% CI: 1.18–2.41; p = 0.004) and marginal (PR; 1.42; 95% CI: 1.01–1.99; p = 0.042) OHL and those in the lower social classes (C-D-E) (PR: 1.85; 95% CI: 1.39–2.47; p<0.001) had more teeth with cavitated carious lesions. In conclusion, adolescents aged 15 to 19 years with poorer levels of OHL had a larger number of teeth with cavitated carious lesions, independently of their socioeconomic status and history of visiting a dentist.

## Introduction

Adolescence is a period of considerable transformations in which individuals often reject predetermined norms and create their own language and behaviors [1]. It can be a vulnerable period in terms of health due to inadequate behavior, the underuse of preventive services and greater independence from one's parents [2,3]. With regard to oral health, the most recent Brazilian epidemiological survey found that 13.6% of adolescents aged 15 to 19 years had never visited a dentist [4].

Previous studies reveal that socioeconomic factors may be associated with dental caries in adolescents [5,6]. Such investigations show that an unfavorable economic status is reflected in a lower brushing frequency, inadequate sanitary installations and the consumption of more cariogenic foods and beverages. This fact makes socioeconomic status an important determinant for the greater prevalence of dental caries [7–9].

The influence of oral health literacy (OHL) on dental conditions has currently piqued the interest of researchers [10,11]. OHL is the ability to understand information and use oral health services for decision making [12]. Studies have demonstrated that OHL exerts an influence on seeking dental services, resulting in a better oral health status. A recent study demonstrated the adequate psychometric capacity of the Brazilian version of the Rapid Estimate of Adult Literacy in Dentistry (BREALD-30) for measuring the level of OHL in Brazilian adolescents [13]. Health literacy is composed of different abilities, such reading, understanding, writing and applying health-related information. The BREALD-30 measures functional oral health literacy through word recognition and reading. These basic skills are important because low functional oral health literacy translates to a limited capacity for understanding oral health information. This is an essential component of oral health literacy that precedes interactional and critical oral health literacy, which are essential domains of decision making [14].

Studies using validated instruments have shown that adolescents with inadequate OHL are more prone to inadequate behavior and practices that place their health at risk, along with medication errors and poor oral hygiene [3,5], which can lead to poor oral health. Thus, an improvement in OHL could contribute to a reduction in disparities regarding oral health in the population [15].

The majority of population-based studies on oral health in Brazil focus on preschoolers and schoolchildren up to 12 years of age, while little is known regarding the oral health of adolescents aged 15 to 19 years [16, 17]. It is in this phase that the onset of preventive attitudes arises in a conscious and intentional manner, when the individual begins to associate oral health with aspects of appearance and prestige and no longer accepts parental supervision [18]. To the best of our knowledge, no studies have investigated the influence of OHL in individuals in this age range.

Therefore, the aim of the present study was to investigate whether the level of OHL is associated with the number of teeth with cavitated carious lesions in adolescents aged 15 to 19 years.

## Material and methods

### Ethical considerations

This study received approval from the Research Ethics Committee of the Paraiba State University (Certificate number: 55953516.2.1001.5187) and followed the guidelines of the Helsinki Declaration. The consent was informed and obtained in writing. Consent was obtained from both the parents and adolescents.

### Study design and sample

An analytical, cross-sectional study was conducted at public and private schools in Campina Grande, which is a medium-sized city in northeast Brazil with a Human Development Index of 0.72.

The planning of this study was based on the guidelines of the Strengthening the Reporting of Observational Studies in Epidemiology (STROBE initiative) [19]. The data were collected between October 2016 and July 2017.

A representative sample was selected using two-stage (schools and adolescents) probabilistic cluster sampling method stratified by administrative district of the city and type of school (public or private). One hundred thirty-one schools were registered with the Ministry of Education. Firstly, 32 schools were selected using a randomization process considering the distribution of adolescents in the six administrative districts of the city. A total of 16 public and 16 private schools were included in the study. Students were then selected at each school using a simple randomization procedure. The final number of students was proportional to the population. The sample size was calculated considering a 5% margin of error, 95% confidence interval, 50% prevalence of caries and a design effect of 1.6. A minimum sample of 641 adolescents was determined, which was increased to compensate for an estimated 20% dropout rate, leading to a final sample of 769 adolescents.

### Eligibility criteria

The inclusion criteria were literate male and female adolescents aged 15 to 19 years attending the school system. Adolescents with learning problems due to medical conditions, syndromes that affect learning abilities and hearing impairment (information obtained from parents and confirmed by teachers) were excluded in the study.

### Training and calibration exercises

The training exercise for the diagnosis of dental caries using the Nyvad Index [20] was conducted by an experienced specialist in two steps based on the method proposed by Peres et al. [21]. In the theoretical step, the criteria for the diagnosis, clinical chart and routine to be followed during the clinical examination were studied. Two examiners also analyzed images of the conditions to be investigated. In the practical step, the examiners were asked to diagnose oral problems. The calibration exercise involved the determination of inter-examiner and intra-examiner agreement using the Kappa statistic (K = 0.89 to 0.90 and 0.88 to 0.90, respectively).

The training and calibration of the two interviewers for the use of the BREALD-30 followed the method proposed by the authors who validated the instrument in Brazil [22], using a video bank of the administration of the instrument to volunteers with different levels of OHL. This phase was coordinated by a researcher with theoretical and practical experience (considered the gold standard) in OHL and the BREALD-30. Kappa coefficients between the examiners and gold standard were 0.889 and 0.884. Agreement between the two examiners was 0.870 and intra-examiner agreement was 0.898 and 0.871. The intraclass correlation coefficient (ICC) was used to evaluate agreement on the total BREALD-30 scores; inter-examiner agreement—ICC = 0.987 (95% CI: 0.970 to 0.995) and 0.874 (95% CI: 0.860 to 0.895); and intra-examiner agreement—ICC = 0.973 (95% CI: 0.921 and 0.991) and 0.994 (95% CI: 0.982 to 0.998). This step was conducted with 50 children in a school chosen by convenience.

### Pilot study

A pilot study was conducted with 50 adolescents at a public and private school. These subjects were selected by convenience and were not included in the main study. The results of the pilot study revealed that there was no need to change the methods.

### Collection of clinical data

The adolescents performed supervised oral hygiene prior to the clinical examinations to facilitate the diagnosis. The participants were examined individually in a reserved room sitting in front of the examiner, who was duly equipped with individual protective equipment, a head lamp (Petzl Zoom; Petzl America, Clearfield, UT, USA), sterilized mouth mirror (PRISMA, São Paulo, Brazil), sterilized Williams probe (WHO-621; Trindade, Campo Mourão, Brazil) and gauze to dry the teeth.

The Nyvad Index [20] was used for the diagnosis of dental caries. This index is based on visual and tactile findings and uses the following classification: (0) sound tooth; (1) carious lesion with surface intact; (2) active lesion with surface interrupted; (3) active lesion with cavitation; (4) inactive lesion with surface intact; (5) active lesion with surface interrupted; (6) inactive lesion with cavitation; (7) restoration in good state; (8) restoration with active lesion; and (9) restoration with inactive lesion. For statistical purposes, dental caries was only considered in the present study when cavitated lesions were found (codes 3 and 6).

### Collection of non-clinical data

Meetings were held with parents to explain the importance of the study. On the occasion, the parents answered a sociodemographic questionnaire addressing the guardian's age, race and sex of the adolescent, mother's schooling, social class, number of residents in the home and birth order of the adolescent. Forty-two years old was chosen as the cutoff for guardian’s age based on the median. The adolescents were asked if they had ever been to a dentist. Social class was categorized using the questionnaire of the Brazilian Economic Classification Criteria based on the possession of consumer goods. This classification defines the following social classes: A1 (highest), A2, B1, B2, C, D and E (lowest) [23].

The BREALD-30 was administered in interview form to measure the OHL of the adolescents. This instrument is the Brazilian version of the Rapid Estimate of Adult Literacy in Dentistry-30 (REALD-30), which is a pioneering questionnaire based on the recognition of words related to dentistry that has been validated in several different languages [15,17]. The BREALD-30 is composed of 30 words related to dentistry organized in a logical sequence with an increasing level of reading difficulty [15]. The adolescents read the 30 words aloud to the interviewer. One point was awarded for each correctly pronounced word and zero was marked when the word was pronounced incorrectly. Thus, a higher number of correctly pronounced words resulted in a higher score, denoting a higher level of OHL.

### Statistical analysis

The organization of the data and the statistical analysis were performed using the Statistical Package for the Social Sciences (SPSS for Windows, version 22.0; IBM Inc., Armonk, NY, USA). The number of teeth with cavitated carious lesions (Nyvad Index) was the dependent variable and was treated as a discrete numeric variable. The codes used to demonstrate the presence of cavitated carious lesions were 3 and 6 on the Nyvard Index.

The independent variables were OHL, sociodemographic characteristics and visit to the dentist. Forty-two years old was chosen as the cutoff for guardian’s age based on the median. Variables with a p-value < 0.20 in the bivariate model were incorporated in the multivariate model using the backward stepwise method. Variables with p < 0.05 in the multivariate model were considered to be significantly associated with the outcome and were maintained in the final model. The total BREALD-30 score was categorized as inadequate, marginal or adequate literacy based on the distribution of the scores: 0–18, 19–22 and 23–30, respectively, using tertiles of the sample distribution as the cutoff points [24].

**Fig 1:**
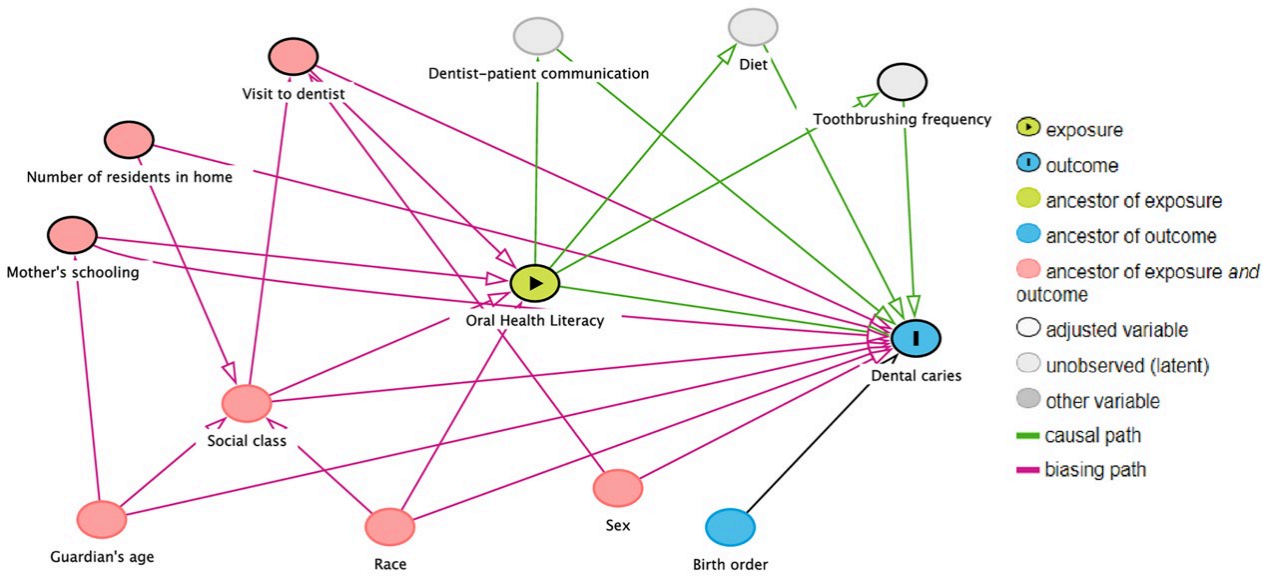
Adopted causal structure of secondary data analysis of dental caries.

A Directed Acyclic Graph (DAG) was used to assist in the selection of covariables for the statistical adjustments and support the causal interpretation of the effect of the exposure on the outcome, as displayed in Fig 1. DAG is an important tool for reducing bias in estimates produced through the selection and adjustment of covariates. The DAG approach involves an exposure (main independent variable) and an outcome (dependent variable). DAG also illustrates other independent variables, ancestors (a direct or indirect cause of a particular variable), adjustment or control variables and latent variables that are not within the scope of the study but are included due to their influence on the outcome [25].

## Results

Seven hundred forty-six adolescents participated in the present study, corresponding to a 97% response rate. Twenty-three adolescents were excluded for failing to appear on the days scheduled for the examinations.

Table 1 displays the characteristics of the sample. The majority was female (59.5%), self-declared as non-white (71.7%), was not the oldest child (57.9%), lived with up to five people in the home (83%), had been to a dentist at least once (93%) and had mothers with more than eight years of schooling (59.4%). 92.8% of the adolescents presented at least one caries lesions in any stages. Cavitated carious lesions were found in 41.6%. Only 29.4% of the adolescents exhibited an adequate level of OHL.

**Table 1:** Characterization of sample.

Variable	n	%
**Sex**		
Female	444	59.5
Male	302	40.5
**Race**		
White	211	28.3
Non-white	535	71.7
**Birth order**		
Youngest child	248	33.2
Middle child	184	24.7
Oldest child	314	42.1
**Visit to dentist**		
Yes	694	93.0
No	46	6.2
**Mother's schooling**		
< 8 years of study	299	40.1
≥ 8 years of study	443	59.4
**Socioeconomic status**		
Lower	428	56.9
Higher	318	42.3
**Guardian's age**		
≤ 42 years	384	51.5
> 42 years	360	48.3
**Number of residents in home**		
1 to 5	619	83.0
6 or more	125	16.8
**Experience of dental caries**		
Yes	692	92.8
No	54	7.2
**Cavitated carious lesions**		
Yes	310	41.6
No	436	58.4
**Recognition ability of oral health terms**		
Inadequate	247	33.1
Marginal	280	37.5
Adequate	219	29.4

In the bivariate analysis, the following variables were associated with cavitated carious lesions: social class, mother's schooling, number of residents in the home, birth order and OHL. In the multivariate analysis, however, the only variables that remained in the final model were OHL and social class. Adolescents with inadequate OHL (PR: 1.69; 95% CI: 1.18 to 2.41; p = 0.004) and marginal OHL (PR: 1.42; 95% CI: 1.01 to 1.99; p = 0.041) and those in the lower social classes (C-D-E) (PR: 1.85; 95% CI: 1.39 to 2.47; p <0.001) had significantly more teeth with cavitated carious lesions (Table 2).

**Table 2:** Poisson regression analyses of variables associated with the number of teeth with cavitated carious lesions in adolescents.

Variable	Number of teeth with cavited carious lesions		Bivariate*		Multivariate**	
	Mean	Std. Deviation	p-value	Unadjusted PR (95% CI)	p-value	Adjusted PR (95%CI)
**Sex**						
Female	0.88	1.35	0.814	1.03 (0.80–1.32)	-	-
Male	0.85	1.57		1.00	-	-
**Socioeconomic status**						
Lower	1.13	1.62	<0.001	2.14 (1.64–2.79)	<0.001	1.85 (1.39–2.47)
Higher	0.53	1.09		1.00	-	1.00
**Mother's schooling**						
< 8 years of study	1.07	1.67	0.002	1.46 (1.15–1.85)	-	-
≥ 8 years of study	0.73	1.26		1.00	-	-
**Guardian's age**						
≤ 42 years	0.86	1.44		1.00	-	-
> 42 years	0.88	1.46	0.841	1.02 (0.80–1.30)	-	-
**Race**						
White	0.74	1.44		1.00	-	-
Non-white	0.92	1.45	0.143	1.24 (0.92–1.67)	-	-
**Number of residents in home**						
1 to 5	0.80	1.39		1.00	-	-
≥ 6	1.16	1.67	0.012	1.44 (1.08–1.92)	-	-
**Birth order**						
Youngest child	0.82	1.44	0.728	1.05 (0.78–1.41)	-	-
Middle child	1.10	1.57	0.017	1.41 (1.06–1.87)	-	-
Oldest child	0.78	1.36		1.00	-	-
**Recognition ability of oral health terms**						
Inadequate	1.15	1.71	<0.001	2.07 (1.47–2.90)	0.004	1.69 (1.18–2.41)
Marginal	0.86	1.32	0.010	1.55 (1.11–2.16)	0.041	1.42 (1.01–1.99)
Adequate	0.56	1.19		1.00	-	1.00
**Visit to dentist**						
No	1.00	1.85	0.599	1.15 (0.67–1.99)	-	-
Yes	0.86	1.42		1.00	-	-

*Poisson regression analysis unadjusted for independent variables and number of teeth with cavitated carious lesions

**Poisson regression analysis adjusted for independent variables and number of teeth with cavitated carious lesions

Variables with p < 0.20 in bivariate analysis incorporated into multivariate model: social class, mother's schooling, number of residents in home, birth order, race and oral health literacy. Controlled for confounding effects according to DAG: social class, mother's schooling and race.

## Discussion

The main findings of the present study demonstrate that, after controlling for confounding variables, lower levels of OHL were associated with an increase in the number of cavitated carious lesions among the adolescents. A practical implication of this result may be the inefficacy of reading materials addressing the prevention of dental caries by adolescents whose ability to recognize oral health terms is limited. To the best of our knowledge, this is the first study to perform this evaluation with adolescents.

The Nyvad Index was used for the diagnosis of dental caries [20]. This index was created to evaluate the development of carious lesions on a scale from sound teeth to cavitated dentinal lesions and can be used in clinical and epidemiological studies [26]. A high prevalence of dental caries was observed when initial caries lesions were considered (92.8%), as occurred in previous studies [17,27]. On the other hand, we opted for analyzing dental caries only as cavitated carious lesions, which constitute a chronic, irreversible stage that exerts a higher impact on dental structures and is the worse outcome of this dental disease. OHL may influence cavitated carious lesions by reducing the occurrence of dental visits and appropriate treatment. The effect of OHL on white spots should be investigated in future studies with a more preventable perspective.

For the purposes of analysis, codes 3 and 6 of the Nyvad present Index were used, which correspond to cavitated lesions. These are the lesions that most often drive the search for treatment. The prevalence was 41.6%, accounting for nearly half of the total prevalence of dental caries in the study. Thus, although the majority of adolescents had been to a dentist at some time in life, they may not have been receiving adequate dental care. Similar results are found in national studies involving the same age group [16,17].

The prevalence of cavitated carious lesions was higher in the present study compared to that reported in studies conducted in more developed countries, where adolescents likely receive more dental care [28,29]. The findings reflect a need for more effective preventive measures among adolescents and the use of dental techniques to limit the harm caused by caries when this process has already begun.

Lower levels of OHL can exert a negative influence on caries prevention and control [30,31]. In the present study, adolescents with inadequate or marginal OHL had a greater number of cavitated carious lesions compared to those with adequate OHL. This finding is worrisome, as adolescents constitute a vulnerable population prone to risk behaviors and often reject pre-determined healthy habits [3,10,31]. Previous studies involving other age groups report similar results, as individuals with a lower level of OHL were found to have greater caries experience and greater treatment needs [32].

In studies involving OHL and the oral health of children, particularly preschoolers, the OHL of the parents/caregivers is measured, since they are responsible for oral health practices in children at this age [33,34]. However, it is necessary to measure the OHL of adolescents, since they have greater autonomy with regard to parents in this period and exhibit changes in oral health interpretations and behaviors [35].

Studies on OHL are necessary, since such investigations go beyond clinical conditions. Recent studies report that a higher level of OHL leads to less expenditures on oral health, fewer missed dental appointments and less anxiety during dental treatment [30,36]. Studies of this type enable the identification of individuals with inadequate OHL, assisting dentists in choosing an adequate language to achieve more effective communication and, consequently, greater assimilation of information related to health [37,38], which would likely result in fewer treatment needs.

The BREALD-30 was used in the present study to measure OHL. This instrument is the Brazilian Portuguese version of the original REALD-30 and has been validated for use on adults and adolescents [12,13]. This is one of the most widely employed instruments in other languages [11,15] as a screening tool for individuals or groups with poorer oral health status [39]. However, its results must be interpreted with caution, as the instrument is used to evaluate the identification of words without testing the comprehension of what is being read. Nonetheless, this instrument is strongly associated with functional literacy and has adequate psychometric properties, making it valid and reliable for measuring this construct [13,40].

Belonging to a lower social class was associated with a greater number of cavitated carious lesions, which is in agreement with data described in previous studies [27,40]. Individuals in lower social classes often have less access to health information, healthy eating, oral health services and personal hygiene products. Consequently, poorer oral health and more caries experience [28,41]

The present findings suggest the need to invest in oral health promotion strategies that increase the level of OHL. This study also draws attention to the need for dentists to improve their use of language when communicating with patients to assist them with decision making in terms of health and the maintenance of healthy habits [2,10,32].

The major limitation of this study is its cross-sectional design, which does not enable establishing cause and effect relationships. However, the validated instruments and representative population-based sample were used to minimize the possibility of biases. Moreover, a DAG with a causal model was used to identify possible confounding factors and explore the influence of socioeconomic and psychosocial factors on the prevalence of cavitated carious lesions. This study used broad inclusion criteria to represent the target population accurately and enhance its external validity. Longitudinal studies are needed for a higher level of evidence regarding the associations found in the present investigation.

## Conclusion

The results of the multivariate analysis revealed that adolescents 15 to 19 years of age with lower levels of oral health literacy had a larger number of teeth with cavitated carious lesions than those with adequate level of OHL, independently of their socioeconomic status and history of visiting a dentist.

## Supporting information

S1 File STROBE checklist. (PDF)

S2 File Database file. (SAV)
